# Pleural Liquid Biopsy for Oncological Practice: A Narrative Review

**DOI:** 10.3390/cancers18172844

**Published:** 2026-09-03

**Authors:** Elisa Roca, Marta Pozzari, Philippe Astoul

**Affiliations:** 1 Thoracic Oncology—Lung Unit, P. Pederzoli Hospital, 37019 Peschiera Del Garda, Italy; 2 Medical Oncology, Azienda Ospedaliero—Universitaria di Cagliari, University of Cagliari, 09042 Cagliari, Italy; m.pozzari@studenti.unica.it; 3 Faculty of Medical and Paramedical Sciences, Aix-Marseille University, 13005 Marseille, France; pastoul@ap-hm.fr

**Keywords:** pleural effusion, liquid biopsy, ctDNA, circulating tumour cells, exosomes, next-generation sequencing, molecular diagnostics, lung cancer, biomarkers

## Abstract

**Background**: Pleural effusion is a common clinical presentation in both benign and malignant conditions. The advent of liquid biopsy allowed new possibilities for non-invasive molecular profiling using body fluids. Pleural fluid, by virtue of its proximity to thoracic malignancies and its rich tumour-derived content, represents a particularly compelling matrix for liquid biopsy analysis. This review synthesises current evidence regarding the diagnostic, predictive, and prognostic utility of pleural liquid biopsy in clinical medicine. **Methods**: A narrative review was conducted using PubMed, MEDLINE, and EMBASE databases. Search terms included combinations of “pleural effusion”, “liquid biopsy”, “circulating tumour DNA”, “circulating tumour cells”, “exosomes”, “next-generation sequencing”, and “biomarkers”. Priority was given to original research articles, systematic reviews, and meta-analyses published between 2013 and 2026. **Results**: Pleural fluid contains a diverse repertoire of tumour-derived analytes, including cell-free and circulating tumour DNA (ctDNA), circulating tumour cells (CTCs), exosomes, and soluble proteins. These biomarkers enable molecular characterisation of underlying malignancies with sensitivity that often exceeds that of plasma-based liquid biopsy and complements histological tissue biopsy. Detection of actionable mutations, including *EGFR*, *ALK*, *KRAS*, and *BRAF* alterations, directly informs targeted therapy selection. Furthermore, serial sampling facilitates real-time monitoring of therapeutic resistance, disease progression, and clonal evolution. **Conclusions**: Pleural liquid biopsy offers a minimally invasive, reproducible, and clinically informative approach to molecular profiling in patients with pleural disease, particularly those with thoracic malignancies. Despite existing challenges in standardisation and analytical sensitivity, its integration into routine clinical pathways holds significant promise for advancing personalised oncological care. Multi-omics integration and artificial intelligence may further consolidate its role in therapeutic decision-making.

## Simple Summary

Pleural effusion frequently occurs in both benign and malignant thoracic disease, and liquid biopsy has emerged as a non-invasive modality for molecular profiling of body fluids. Given its proximity to thoracic malignancies, pleural fluid constitutes a particularly rich matrix for tumour-derived analytes, including cell-free and circulating tumour DNA, circulating tumour cells, exosomes, and soluble proteins. This narrative review synthesises evidence published between 2013 and 2025 on the diagnostic, predictive, and prognostic utility of pleural liquid biopsy. Findings indicate that pleural fluid analysis frequently achieves sensitivity exceeding that of plasma-based liquid biopsy and complements histological assessment, enabling detection of actionable alterations such as *EGFR*, *ALK*, *KRAS*, and *BRAF* mutations to guide targeted therapy selection. Serial sampling further allows real-time monitoring of clonal evolution, therapeutic resistance, and disease progression. Despite persisting challenges in standardisation and analytical sensitivity, pleural liquid biopsy holds substantial promise for advancing personalised oncological management.

## 1. Introduction

Pleural effusion, defined as the pathological accumulation of fluid within the pleural space, represents one of the most frequently encountered clinical syndromes in respiratory and general internal medicine. It is estimated that more than 1.5 million new cases of pleural effusion are diagnosed annually in the United States alone, with comparable or higher burdens documented in Europe and Asia, which leads to high healthcare costs [1]. Causes range broadly from transudative processes such as cardiac failure and hypoalbuminaemia to exudative aetiologies including parapneumonic effusion, tuberculosis, and, critically, malignancy. Malignant pleural effusion (MPE) accounts for approximately 40–50% of all exudative effusions in adults, with lung cancer, breast cancer, and lymphoma constituting the most common primary sources [2].

The clinical importance of pleural effusion extends beyond symptomatic palliation. Its aetiological characterisation has direct implications for prognosis and therapeutic management, particularly in the oncological setting. Conventionally, the diagnostic workup relies on cytological analysis of pleural fluid and, where indicated, pleural tissue biopsy obtained via thoracoscopy or image-guided needle biopsy. However, cytology yields a definitive malignant diagnosis in only 60–70% of cases, and tissue acquisition is not always feasible due to patient performance status, anatomical constraints, or insufficient tissue adequacy for comprehensive molecular analysis [3].

Against this backdrop, the concept of liquid biopsy has emerged as a transformative paradigm in oncology. Liquid biopsy refers to the sampling and analysis of non-solid biological materials, most commonly blood, but increasingly urine, cerebrospinal fluid, and pleural fluid, to detect and characterise tumour-derived molecular signals. The principal analytes include cell-free DNA (cfDNA) harbouring tumour-specific mutations, circulating tumour DNA (ctDNA), circulating tumour cells (CTCs), tumour-derived extracellular vesicles (exosomes), and tumour-educated platelets [4].

Pleural fluid occupies a unique niche within the liquid biopsy landscape. Unlike peripheral blood, which receives tumour-derived material following systemic dissemination, pleural fluid is in direct anatomical continuity with pleural and pulmonary tumours. This proximity results in markedly elevated concentrations of tumour-derived nucleic acids and cellular components compared to contemporaneous plasma samples, potentially affording superior diagnostic sensitivity [5]. Furthermore, therapeutic thoracocentesis, routinely performed for symptomatic relief, provides an opportunity to collect biologically informative material without additional procedural risk to the patient.

This narrative review aims to provide a comprehensive and critically appraised synthesis of current evidence on pleural liquid biopsy. Specifically, it addresses the biological basis of tumour-derived analytes in pleural fluid, the diagnostic performance of pleural liquid biopsy relative to conventional methods, its predictive and prognostic applications, its role in clinical decision-making, and the challenges and future directions that will shape its integration into routine practice.

This review builds upon and expands on previous work in this field; in particular, the 2021 review by Sorolla and colleagues catalogues circulating tumour cells detectable in pleural fluid, extracellular vesicles, tumour-derived nucleic acids and the technical platforms used for their detection [6]. The proposed review is notable for three aspects: it extends the literature review up to 2026; it goes beyond the simple cataloguing of diagnostic biomarkers by systematically addressing prognostic and predictive applications related to clinical decision-making in various types of cancer; it incorporates emerging perspectives on artificial intelligence and multi-omics, carrying out a structured comparison between pleural fluid biopsy and tissue- and plasma-based liquid biopsies—aspects that had not previously been addressed [6,7].

## 2. Methods

The narrative review we are presenting was conducted by searching PubMed, MEDLINE, and EMBASE for articles published between 1 January 2013 and 24 August 2026, using combinations of these search terms: “liquid biopsy”, “pleural effusion”, “circulating tumour cells”, “circulating tumour DNA”, “exosomes”, “next-generation sequencing”, and “biomarkers”. Reference lists of retrieved articles and relevant prior reviews were hand-searched to identify additional eligible studies. Systematic reviews and meta-analyses, in addition to original research articles, published in English that reported on tumour-derived analytes in pleural fluid, or that directly compared pleural fluid with tissue or plasma-based liquid biopsy, were eligible for inclusion. Conference abstracts without an associated full-text publication, case reports describing fewer than five patients, and studies restricted to preclinical or animal models without translational relevance to human pleural disease were excluded. Titles and abstracts were first screened for relevance, followed by full-text review of potentially eligible articles; given the narrative, rather than systematic, design of this review, formal risk-of-bias assessment and quantitative synthesis were not performed.

## 3. Biological Basis of Pleural Liquid Biopsy

### 3.1. Composition of Pleural Fluid

Normal pleural space contains a small volume (approximately 0.1–0.2 mL/kg) of ultrafiltrate with low protein content, facilitating frictionless lung movement. The onset of pleural effusion is the result of dysregulation of transpleural pressure balance due to a deficit of both the lymphatic drainage and liquid absorption and increased endothelial permeability [8]. In the setting of malignancy or inflammation, this composition is profoundly altered. Pathological pleural fluid contains a complex milieu of cellular and acellular components that reflect the underlying disease process [9].

Cell-free DNA (cfDNA) is released into the pleural space through multiple mechanisms, including necrosis, apoptosis, and active secretion by tumour cells. Within the cfDNA pool, circulating tumour DNA (ctDNA), which carries tumour-specific somatic mutations, copy number alterations, and epigenetic modifications, constitutes the subset of greatest analytical interest. The concentration of ctDNA in pleural fluid has been reported to be 10- to 100-fold higher than that in matched plasma samples, a finding consistently replicated across studies in lung, breast, and mesothelioma [10].

CTCs represent intact viable tumour cells shed into the fluid compartment. Their detection provides opportunities for morphological characterisation, immunophenotyping, and ex vivo functional assays. Although CTC enumeration in pleural fluid remains technically challenging due to the high background of mesothelial and inflammatory cells, advances in microfluidic and immunomagnetic isolation platforms have improved yield [11].

Exosomes and other extracellular vesicles (EVs) are nanosized membrane-bound particles (30–150 nm) secreted by tumour cells into the pleural space. They carry a molecular cargo of nucleic acids (including microRNAs, long non-coding RNAs, and DNA), proteins, and lipids that reflect the proteogenomic characteristics of their cell of origin. Tumour-derived exosomes in pleural fluid have been implicated in local immunosuppression, promotion of mesothelial-to-mesenchymal transition, and intercellular communication within the tumour microenvironment [12].

In addition to nucleic acid-based analytes, pleural fluid contains a rich proteomic landscape including growth factors, cytokines, angiogenic mediators, and shed tumour antigens. Proteins such as mesothelin, fibulin-3, vascular endothelial growth factor (VEGF), and cancer antigen 125 (CA-125) have been investigated as diagnostic and prognostic biomarkers [13].

### 3.2. Mechanisms of Tumour-Derived Material Release

**Figure 1:**
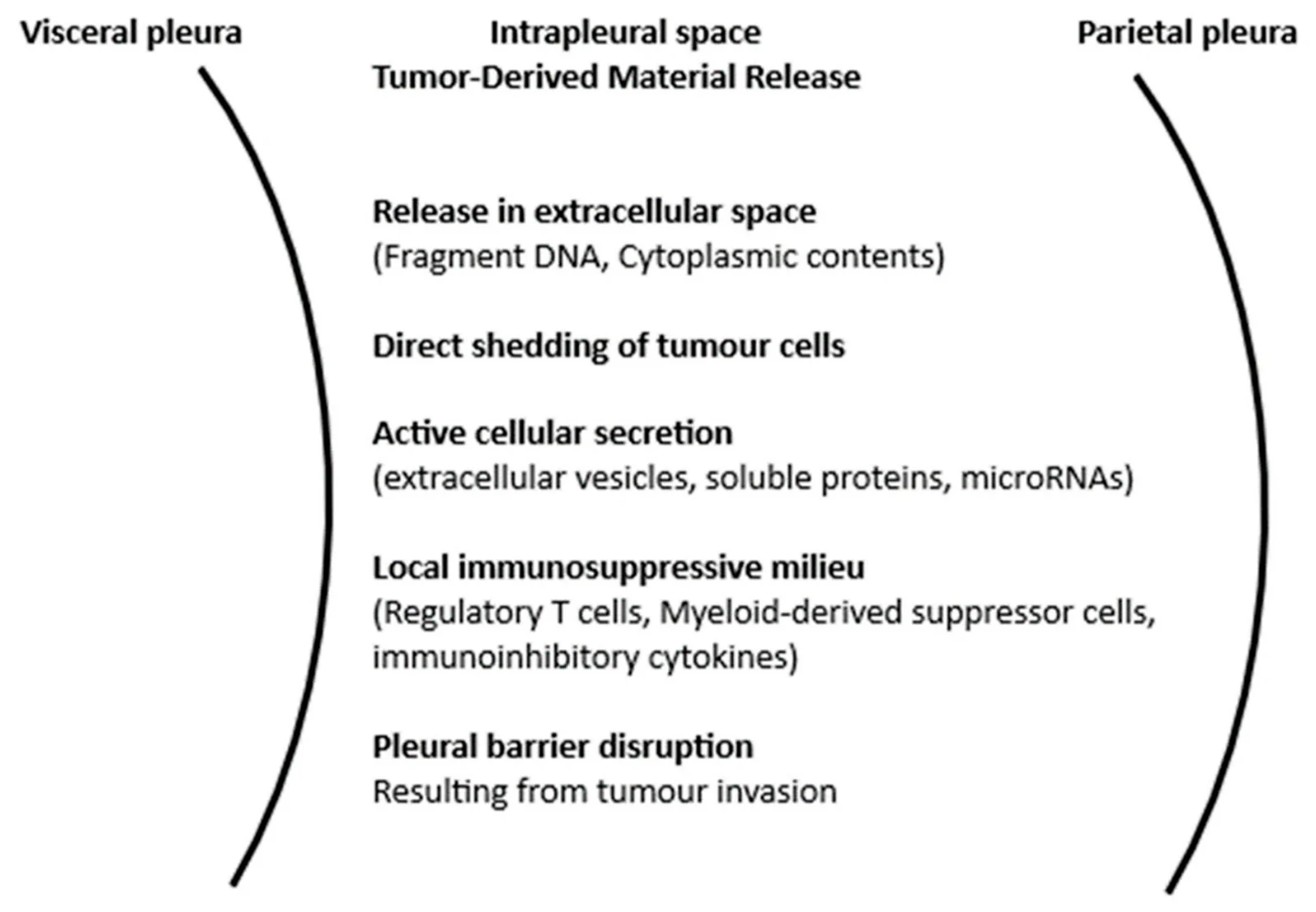
Mechanism of intrapleural tumour-derived material release.

The accumulation of tumour-derived material in the pleural space is governed by multiple, partially overlapping mechanisms that are summarized in Figure 1. Necrotic and apoptotic cell death, particularly common in rapidly proliferating, poorly vascularised tumour cores, releases large quantities of fragmented DNA and cytoplasmic contents into the adjacent extracellular space. These fragments diffuse or are transported lymphatically into the pleural cavity [14].

Active cellular secretion represents a second major pathway. Viable tumour cells at the pleural surface or within pleural nodular deposits continuously secrete EVs, soluble proteins, and microRNAs as part of their intercellular communication repertoire. The pleural fluid, with its limited clearance mechanisms compared to the vascular compartment, serves as an accumulation reservoir for these secreted materials, further amplifying their detectable concentration [15].

Direct shedding of tumour cells, either from primary pleural involvement or from haematogenous metastatic deposits, contributes to the CTC pool within pleural fluid. Pleural barrier disruption resulting from tumour invasion enhances bidirectional exchange between the pleural space and the subpleural vasculature, facilitating both the entry of circulating malignant cells and the efflux of pleural fluid-derived signals into the systemic circulation [16].

A final mechanism of particular relevance to immunological and exosomal studies is the active immunomodulatory role of the tumour microenvironment within the pleural space. Pleural tumour deposits engender a local immunosuppressive milieu rich in regulatory T cells, myeloid-derived suppressor cells, and immunoinhibitory cytokines, a context that shapes the molecular phenotype of EVs and soluble mediators present in the effusion [17].

## 4. Diagnostic Applications

### 4.1. Pleural Liquid Biopsy Versus Traditional Tissue Biopsy

The gold standard for diagnosing malignant pleural effusion and characterising the underlying tumour has historically been cytological examination of pleural fluid combined, where necessary, with histological analysis of pleural tissue obtained by closed needle biopsy, CT-guided biopsy, or thoracoscopy. While these methods retain an essential role, they are limited by several factors: cytology is insufficiently sensitive, tissue biopsy carries procedural risk, especially in patients frequently presenting multiple comorbidities, and both approaches are subject to spatial and temporal tumour heterogeneity [18].

Pleural liquid biopsy addresses several of these limitations. Firstly, it is accessible through a procedure (thoracocentesis) that is routinely performed for diagnostic and therapeutic purposes, requiring no additional invasive steps. This results in a relevant reduction in patient morbidity and costs. Secondly, the molecular analysis of pleural fluid ctDNA interrogates a broader genomic profile than a single-site tissue biopsy, potentially capturing intratumoural heterogeneity [19].

Multiple studies have directly compared the diagnostic yield of pleural ctDNA analysis with contemporaneous cytology or tissue biopsy. A landmark study by Yanagita et al. (2016) demonstrated that *EGFR* mutation detection in pleural fluid ctDNA achieved a sensitivity of 92% and specificity of 96% in patients with known EGFR-mutant lung adenocarcinoma, compared with 65% sensitivity for cytology alone in the same cohort [20]. Comparable findings have been reported for *KRAS*, *BRAF*, and *ALK* alterations, with pleural fluid consistently outperforming plasma-based cfDNA profiling.

### 4.2. Pleural Liquid Biopsy Versus Traditional (Plasma-Based) Liquid Biopsy

**Figure 2:**
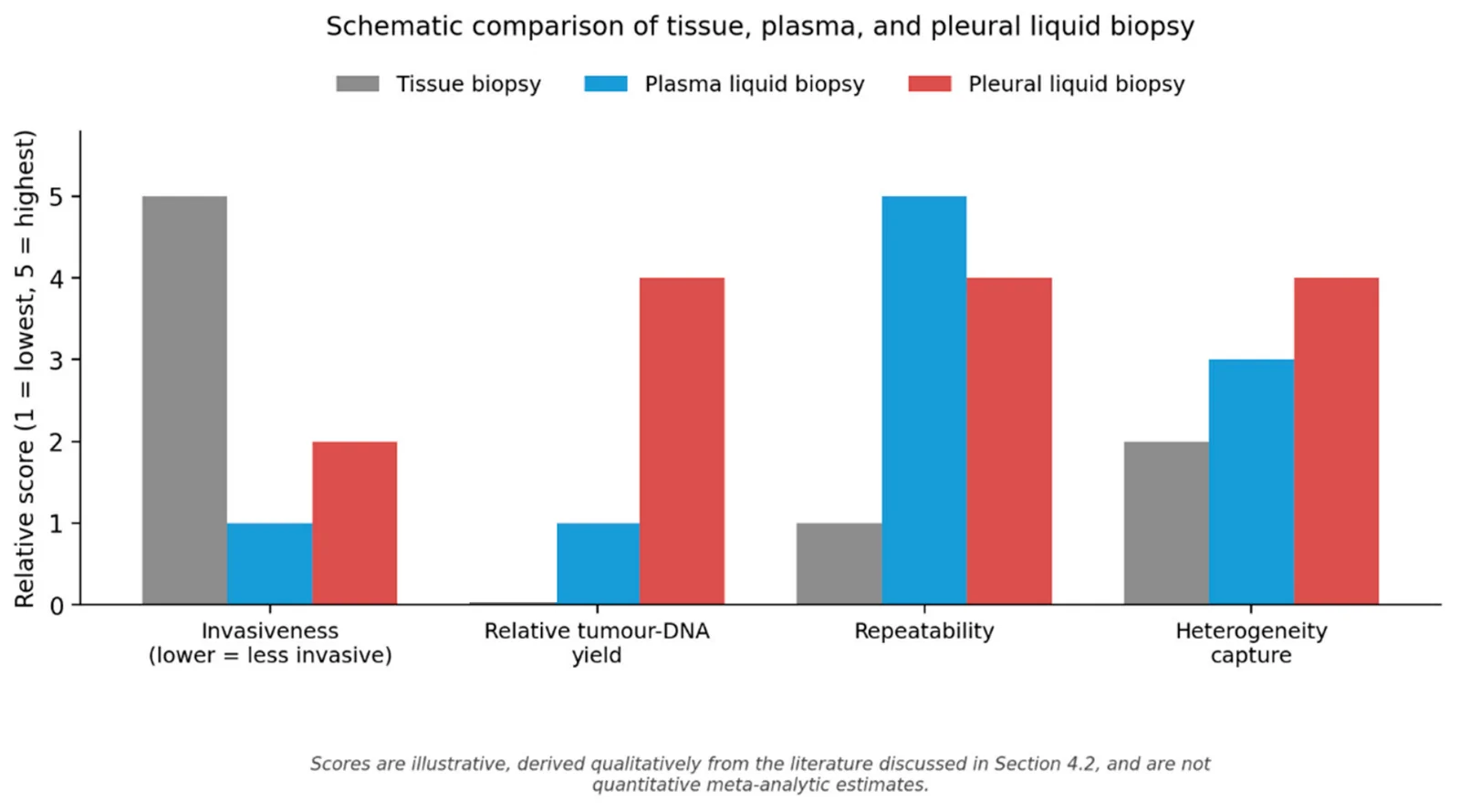
Schematic comparison of tissue biopsy, plasma-based liquid biopsy, and pleural liquid biopsy across key operational and analytical dimensions.

Peripheral plasma has been the most widely studied substrate for liquid biopsy in oncology, and pleural fluid is frequently discussed only as one of several ‘non-plasma’ alternatives, alongside cerebrospinal fluid, urine, and ascites [7]. Direct comparison of these three sampling strategies—tissue biopsy, plasma, and pleural fluid—defines where pleural liquid biopsy determines a distinct advantage. Tissue biopsy remains the gold standard for histological confirmation and captures the full architecture of the tumour, but it is invasive, it cannot always be repeated, and it is subject to sampling bias from a single anatomical site. Instead, plasma-based liquid biopsy is minimally invasive and widely accessible, but ctDNA concentrations are frequently at or below the limit of detection of available assays, particularly in early-stage or low-burden disease. Pleural fluid occupies an intermediate position: the procedure for obtaining it is only marginally more invasive than venepuncture in patients who already require thoracocentesis for symptom relief; however, owing to its direct anatomical continuity with pleural and pulmonary tumours, it typically contains substantially higher concentrations of tumour-derived nucleic acids than matched plasma [10]. A comparative overview of these three matrices is presented in Table 1 and Figure 2.

### 4.3. Sensitivity, Specificity, and Diagnostic Yield

The diagnostic performance of pleural liquid biopsy varies according to the analyte interrogated, the tumour type, the volume of effusion analysed, and the technological platform employed. Overall, studies report sensitivities ranging from 75% to 95% for mutation detection using PCR-based methods, and up to 97% with comprehensive next-generation sequencing (NGS) panels, when compared to tissue-confirmed diagnoses [22].

Gene-specific concordance data further illustrate this performance. The importance of pleural fluid is emphasised when tissue is insufficient or unavailable for comprehensive molecular analysis [23]. Regarding *EGFR* mutation, concordance rates between pleural ctDNA and matched tumour tissue typically exceed 85%, with discordant cases often attributable to evolution under selective treatment pressure or spatial heterogeneity [24]. *ALK* rearrangements, obtained by fluorescence in situ hybridisation, immunocytochemistry on cytospin preparations, or RNA-based NGS assays targeting fusion transcripts, could be identified in pleural fluid with a sensitivity ranging from 70% to 88% and a specificity exceeding 95% across published series [25]. In pleural fluid, other mutation ctDNA with high concordance to matched tumour tissue has likewise been detected: *KRAS G12C* and *BRAF V600E* [26].

In malignant pleural mesothelioma, the reported diagnostic sensitivity of cytological samples is about 75% and is currently not completely satisfying. However, some authors recently reached a diagnostic sensitivity up to 95%, exploring the application of nanostring technology in this field [27,28].

An important consideration is the distinction between diagnostic (aetiological) and genotypic (mutation detection) sensitivity. For the former, establishing that an effusion is of malignant origin, ctDNA analysis alone may be insufficient, as benign inflammatory processes can generate cfDNA that mimics certain non-specific signatures. Integration with cytological, biochemical (LDH, protein ratio), and immunological markers is essential for a complete diagnostic framework [29].

For genotypic analysis in confirmed malignancy, the specificity of targeted mutation detection in pleural fluid approaches 98–100%, as driver mutations such as *EGFR* exon 19 deletions and L858R substitutions are, by definition, absent in non-malignant cells. The principal source of false negatives is insufficient ctDNA input, which may arise from effusions with low tumour cell burden or from early-stage disease [30].

### 4.4. Technological Platforms

The primary technologies employed for pleural liquid biopsy analysis can be broadly divided into PCR-based and sequencing-based approaches. Digital PCR (dPCR), including droplet digital PCR (ddPCR), offers exceptional sensitivity for the detection of known, pre-specified mutations at variant allele frequencies as low as 0.01%, making it well-suited for monitoring residual disease and early resistance emergence [31]. However, its utility is limited to pre-selected targets and it cannot identify novel or unexpected alterations [25].

Next-generation sequencing (NGS) platforms, by contrast, enable simultaneous interrogation of hundreds of genomic loci, providing comprehensive mutational profiling including single nucleotide variants, insertions and deletions, copy number variations, and structural rearrangements. Panel-based NGS (e.g., Foundation One Liquid, Guardant360, and custom institutional panels) has become the preferred approach in centres with the requisite infrastructure. Whole-exome and whole-genome sequencing remain predominantly research tools but are increasingly informing translational discoveries [32].

Emerging platforms include methylation-based assays, which interrogate tumour-specific epigenetic patterns in cfDNA to infer tissue of origin, a capability of particular relevance in pleural effusions of unknown primary. Multi-analyte approaches that simultaneously profile ctDNA, exosomal RNA, and protein markers are under active investigation and hold promise for enhancing diagnostic accuracy beyond what any single analyte can achieve [33].

## 5. Predictive and Prognostic Value

### 5.1. Identification of Actionable Mutations

One of the most clinically impactful applications of pleural liquid biopsy is the detection of actionable driver mutations that direct targeted therapy. NGS analysis and whole-exome sequencing might reveal the mutational heterogeneity landscape that could be incompletely defined in a portion of tissue biopsies, providing additional information and the development of novel target therapies [34].

In lung adenocarcinoma—which accounts for the majority of MPEs—a substantial proportion of tumours harbour alterations in *EGFR*, *ALK*, *ROS1*, *MET*, *RET*, *KRAS*, or *BRAF*, many of which are targeted by approved or investigational therapies. The ability to identify these alterations from pleural fluid has direct implications for treatment planning, particularly in the diagnostic settings discussed in Section 4.3 [23].

*EGFR* mutations are the most extensively characterised in the pleural liquid biopsy literature. Multiple prospective studies have confirmed that EGFR mutation detection in pleural fluid ctDNA is feasible and clinically concordant with tissue-based results, a diagnostic performance discussed in detail in Section 4.3 [24]. Serial pleural ctDNA analysis could identify *EGFR T790M* resistance mutations caused by treatment pressure, a monitoring application addressed in Section 5.2.

*ALK* gene rearrangements, although less amenable to detection by standard PCR due to their complex structural nature, have been identified in pleural fluid using fluorescence in situ hybridisation (FISH), immunocytochemistry on cytospin preparations, and RNA-based NGS assays targeting fusion transcripts; the corresponding diagnostic sensitivity and specificity are summarised in Section 4.3 [25].

*KRAS G12C* mutations, now targetable with sotorasib and adagrasib, have also been detected in pleural fluid ctDNA, as have *BRAF V600E* mutations amenable to *BRAF/MEK* inhibitor combinations (concordance data summarised in Section 4.3) [26]. The ability to perform comprehensive panel testing from a single pleural fluid specimen thus enables simultaneous detection of multiple actionable targets in a single assay, streamlining the diagnostic pathway.

### 5.2. Therapy Selection and Resistance Monitoring

Beyond initial mutation profiling, pleural liquid biopsy offers a dynamic window into tumour evolution under therapeutic pressure. Because thoracocentesis is often repeated over the course of disease for symptomatic relief, serial sampling of pleural fluid is feasible to monitor disease evolution over time [19], and, in conjunction with longitudinal plasma cfDNA monitoring at disease progression, enables characterisation of acquired resistance mechanisms in real time, an approach analogous to the serial ctDNA surveillance strategies now validated for risk-stratified monitoring in lung cancer [35]. This information is clinically actionable: pleural ctDNA analysis has identified *EGFR T790M* resistance mutations, the principal mechanism of acquired resistance to first- and second-generation EGFR tyrosine kinase inhibitors (TKIs), at progression on first-line erlotinib or gefitinib, directing timely transition to osimertinib (third-generation TKI) [20,24]. Detection of *MET* amplification and emergence of *KRAS* bypass-pathway activation are additional resistance mechanisms well characterised in plasma-based monitoring that, in principle, could similarly be interrogated in pleural fluid to guide enrolment in appropriate clinical trials, although pleural-fluid-specific validation of these particular alterations represents a priority for future study because evidence remains limited.

In mesothelioma, a pleural-predominant malignancy for which tissue acquisition is particularly challenging and for which immunotherapy has emerged as a first-line option, *BAP1* loss and other epigenetic alterations detectable in pleural fluid ctDNA are under investigation as predictive biomarkers for immunotherapy response [36]. Similarly, in breast cancer with pleural metastases, ESR1 mutation detection in pleural ctDNA correlates with endocrine therapy resistance, informing transitions to fulvestrant or CDK4/6 inhibitor combinations [37].

### 5.3. Prognostic Biomarkers

Beyond mutation detection, several quantitative and qualitative features of pleural fluid composition have been associated with patient prognosis. Higher concentrations of ctDNA, reflecting elevated tumour burden, have been associated with shorter overall survival and progression-free survival in lung cancer patients with MPE in some series [38], consistent with the relationship between clinical outcome and tumour genomic heterogeneity [39]. Similarly, high CTC counts in pleural fluid have been correlated with visceral metastatic burden and inferior outcomes.

Exosomal microRNA signatures extracted from extracellular vesicles isolated in pleural fluid have emerged as an area of active investigation. Malignant pleural fluid is richer than plasma in tumour-derived products including micro RNAs (miRNAs), including miR-21, miR-155, and miR-141, distinguishing MPE from benign transudates. The levels are inversely correlated with survival in the malignant subset [6]. Soluble proteins including VEGF and angiopoietin-2 in pleural fluid have been associated with pleural reaccumulation rates and patient prognosis, with high VEGF concentrations predicting early effusion recurrence and supporting consideration of intrapleural bevacizumab [40].

## 6. Clinical Decision-Making

### 6.1. Overview of Clinical Applications

Liquid biopsy can be used at various stages throughout the entire cancer care pathway, from screening at-risk individuals through to initial diagnosis, monitoring of minimal residual disease or recurrence, identifying the causes of acquired drug resistance, and integration into routine clinical practice. Table 2 summarises representative applications across these domains, together with illustrative biomarkers and the corresponding evidence discussed throughout this review.

### 6.2. Clinical Scenarios

The integration of pleural liquid biopsy into clinical practice has the potential to substantially reshape management algorithms for patients presenting with pleural effusion in the context of known or suspected malignancy. Three principal scenarios illustrate its impact.

In the patient with a new pleural effusion and no prior cancer diagnosis, pleural liquid biopsy, performed at the time of diagnostic thoracocentesis, can simultaneously establish the malignant nature of the effusion (through cytology and ctDNA analysis) and provide molecular profiling that guides first-line systemic therapy selection. This parallel diagnostic-and-genotyping approach compresses the time from presentation to treatment initiation, which is particularly valuable in patients with advanced disease and high symptom burden [41].

In the patient with established lung cancer who develops a new or enlarging pleural effusion during therapy, pleural ctDNA analysis can distinguish disease progression from other causes (parapneumonic effusion, treatment-related pleuritis), characterise the molecular landscape of the evolving tumour, and detect resistance-conferring mutations that mandate therapeutic changes. This avoids the delays and risks associated with repeat tissue biopsy in a frequently compromised patient [42].

In patients being considered for intrapleural therapies, such as talc pleurodesis, intrapleural fibrinolytic therapy, or investigational intrapleural immunotherapy, pleural fluid biomarker analysis can inform candidate selection [43].

In addition to these scenarios, the liquid biopsy approach could reduce the psychological burden on both patients and doctors by minimising the number of invasive procedures and diagnostic uncertainty, an outcome dimension increasingly recognised in the broader liquid biopsy literature [44].

## 7. Advantages and Limitations

### 7.1. Advantages

The principal advantage of pleural liquid biopsy lies in its minimally invasive nature. Thoracocentesis is a well-tolerated outpatient procedure with a complication rate below 3% when performed under ultrasound guidance, making repeat sampling feasible throughout the disease course [45]. This repeatability enables longitudinal molecular monitoring, a capability not practically achievable with tissue biopsy.

The superior analyte concentration in pleural fluid compared to plasma is a critical advantage, with ctDNA concentrations documented to be 10–100-fold higher in pleural fluid in multiple paired analyses. This translates into greater analytical sensitivity, particularly for detecting low-frequency mutations and in cases where plasma ctDNA is below the limit of detection of available assays [38].

Additionally, pleural liquid biopsy captures spatial tumour heterogeneity by sampling shed material from multiple tumour foci within the pleural space, potentially providing a more representative molecular snapshot than a single-site tissue biopsy. This is particularly relevant in tumours characterised by high intratumoral heterogeneity, such as mesothelioma and advanced lung adenocarcinoma with multiple pleural nodules [46].

The main advantages of pleural liquid biopsy are summarized in Table 3.

### 7.2. Limitations and Challenges

Despite these advantages, several important limitations must be acknowledged. Foremost among these is the absence of standardised pre-analytical protocols for pleural fluid ctDNA extraction and analysis. Variables including centrifugation conditions, storage temperature, time to processing, and extraction kit selection all influence analyte yield and quality, and currently no universally accepted guidelines exist. This variability hampers inter-institutional comparability and complicates the clinical translation of research findings [47].

False negative results represent a clinically significant limitation, particularly in patients with low-volume or early pleural disease. In up to 20–25% of cases, pleural ctDNA may be undetectable even in the presence of confirmed malignant effusion, likely reflecting low tumour burden, predominantly necrosis-independent cell death, or poor DNA integrity [48]. In these scenarios, complementary assays, including CTC analysis, exosomal RNA profiling, or conventional cytology, are necessary.

The interpretation of pleural cfDNA findings requires caution in the context of clonal haematopoiesis of indeterminate potential (CHIP), a common age-related phenomenon in which somatic mutations arise in haematopoietic progenitor cells and are shed into the cfDNA pool, potentially simulating tumour-derived mutations [49]. Differentiation between CHIP-derived and tumour-derived variants requires matched white blood cell sequencing, which adds complexity and cost to the analytical workflow.

Finally, the lack of prospective randomised controlled trial data demonstrating that pleural ctDNA-directed therapy decisions improve patient outcomes, compared to conventional diagnostic pathways, remains an important evidence gap. While observational and retrospective data are compelling, prospective validation is required before universal adoption can be recommended [50].

Moreover, a recent review about factors influencing the implementation of liquid biopsy in cancer management identified pre-analytical standardisation, laboratory and personnel requirements, and regulatory and policy frameworks as the main cross-cutting barriers to clinical adoption, all of which apply directly to pleural fluid-based testing [51].

## 8. Future Perspectives

### 8.1. Multi-Omics Integration

The next phase of pleural liquid biopsy development will likely be characterised by the integration of multiple molecular analytes within unified analytical frameworks. Multi-omics approaches that simultaneously profile ctDNA mutations, copy number alterations, DNA methylation patterns, exosomal microRNA expression, and proteomic signatures from a single pleural fluid sample are technically feasible and have demonstrated additive diagnostic value in preliminary studies. Integration of these layers provides a more complete molecular portrait of the tumour and its microenvironment than any single analyte alone [52].

Epigenome-wide methylation profiling of pleural cfDNA represents a particularly promising frontier. Methylation signatures are tumour-type-specific, enabling tissue-of-origin classification in MPE of unknown primary, a clinically common and diagnostically challenging scenario. Recent studies using plasma methylation sequencing (e.g., GRAIL Galleri assay, CancerSEEK) suggest high accuracy for multi-cancer detection; application of analogous approaches to pleural fluid, where signal-to-noise ratios are expected to be even more favourable, is an active area of investigation [53].

### 8.2. Artificial Intelligence and Machine Learning

The complexity and high dimensionality of multi-omic pleural fluid datasets necessitate analytical approaches beyond traditional statistical methods. Machine learning (ML) and deep learning (DL) algorithms are increasingly being applied to integrate heterogeneous pleural fluid biomarker data for diagnostic classification, survival prediction, and therapy response modelling. Convolutional neural networks applied to digital cytopathology images of pleural fluid specimens have demonstrated diagnostic accuracies exceeding those of experienced cytopathologists in preliminary studies [54].

Natural language processing (NLP) tools that extract structured molecular information from pleural fluid NGS reports and integrate them with electronic health record data to generate automated therapy recommendations represent an emerging application. These tools, embedded within clinical decision support systems, could significantly reduce the time from pleural fluid analysis to actionable therapeutic guidance, particularly in centres with limited oncological subspecialty access [55].

### 8.3. Intrapleural Drug Delivery and Companion Diagnostics

An underexplored application of pleural liquid biopsy lies in its potential to inform intrapleural drug delivery strategies. Intrapleural therapeutical approaches (immunotherapy, CAR-T cell therapy, and nanoparticle-based drug delivery systems) are explored in early clinical trials for their potential role as regional immune modulators that could complement systemic treatments [56]. Real-time molecular profiling of the pleural immune microenvironment via liquid biopsy will be essential for patient selection and response assessment [21]. Pleural ctDNA and exosomal PD-L1 expression, for instance, may serve as companion diagnostic markers guiding intrapleural anti-PD-1 therapy.

The development of validated companion diagnostics, biomarker assays co-developed and approved alongside specific targeted therapies, for pleural fluid represents a regulatory and commercial priority. Regulatory agencies including the FDA and EMA have begun to issue guidance frameworks for liquid biopsy companion diagnostics, and pleural fluid-specific assays may follow the precedent established for plasma-based platforms [57].

### 8.4. Personalised Medicine

Ultimately, the trajectory of pleural liquid biopsy research converges on the vision of personalised, molecularly guided medicine for patients with pleural disease. The ability to rapidly and repeatedly characterise the molecular phenotype of a tumour from a routinely obtained pleural fluid specimen, guiding therapy selection, monitoring response, detecting resistance, and informing enrolment in precision oncology trials, represents a paradigm shift from anatomy-based to biology-based clinical decision-making. Realising this vision will require harmonised pre-analytical standards, robust analytical validation, prospective clinical evidence, and integrated informatic infrastructure [58,59,60].

## 9. Conclusions

Pleural liquid biopsy has emerged as a clinically promising and scientifically well-supported approach to the molecular characterisation of pleural disease. The pleural space is a biologically privileged site for tumour-derived analyte accumulation, offering ctDNA, CTC, exosomal, and proteomic signals of superior concentration and richness compared to peripheral blood. Current evidence supports the use of pleural ctDNA analysis as a complement, and, in select circumstances, an alternative, to tissue biopsy for the detection of actionable mutations and the monitoring of therapeutic resistance in patients with malignant pleural effusions.

The clinical implications are substantial: integration of pleural liquid biopsy into diagnostic algorithms can compress the time to molecularly informed therapy initiation, provide longitudinal insight into tumour evolution, and guide both systemic and intrapleural treatment decisions. Nonetheless, the field is constrained by the absence of standardised pre-analytical protocols, limited prospective randomised evidence, and interpretive challenges arising from CHIP-derived variants and false negatives in low-burden disease.

Looking forward, the convergence of multi-omics profiling, artificial intelligence-driven data integration, and companion diagnostic development will progressively expand the clinical utility of pleural liquid biopsy. Investigators and clinicians alike are encouraged to participate in collaborative, prospectively designed studies that will generate the evidence necessary to establish pleural liquid biopsy as a standard-of-care tool in the management of pleural malignancy. The pleural space, long regarded as merely a diagnostic terminus, is increasingly recognised as a dynamic molecular window into thoracic oncology.

## Figures and Tables

**Table 1:** Comparison of tissue biopsy, plasma-based liquid biopsy, and pleural liquid biopsy.

Characteristics	Tissue Biopsy	Plasma Liquid Biopsy	Pleural Liquid Biopsy
Invasiveness	High	Low	Moderate
	* often requiring sedation		
Relative tumour-DNA yield	Not applicable	Low	High [8]
Repeatability	Low	High	Moderate-high
	* often impractical		* in recurrent effusion
Applicability	Moderate	High	Moderate
	* accessible lesion and adequate Performance Status is required	* universally applicable	* presence of a pleural effusion is required
Capture of tumour heterogeneity	Single-site snapshot	Systemic	Regional
		* But signal is diluted	* Reflecting pleural tumour deposits
Complementary role	Reference standard for histology	First-line when no effusion is present	Preferred when an effusion is present and tissue is limited or unavailable [17,21]; Symptomatic relief

**Table 2:** Overview of clinical applications of pleural liquid biopsy.

Application Category	Description	Representative Biomarkers/Approaches	Section
Diagnosis	Establishing the malignant origin of an effusion, including in cytology-negative cases	ctDNA (*EGFR*, *KRAS*, *ALK*, *BRAF*), CTC enumeration, exosomal miRNA panels	Section 4
Screening	Identifying thoracic malignancy in patients with a new, otherwise unexplained effusion and tissue-of-origin classification in MPE of unknown primary	Methylation signatures, multi-analyte panels	Section 8.1
Minimal residual disease/recurrence monitoring	Detecting subclinical disease after definitive local therapy or during surveillance	Serial ctDNA quantitation, digital PCR	Section 5.2 and Section 8.1
Cancer drug resistance	Identifying resistance-conferring alterations at disease progression	*EGFR* T790M/C797S, *MET* amplification, *KRAS* bypass-pathway activation, *ESR1* mutations	Section 5.2
Clinical care/decision-making	Guiding first-line therapy selection, informing candidacy for intrapleural therapies, and supporting patient-centred discussions	Actionable-mutation panels, PD-L1 expression, BAP1 status	Section 6.2 and Section 8.3

**Table 3:** Advantages of pleural liquid biopsy.

Advantages of Pleural Liquid Biopsy
Minimally invasive procedure More feasible and easily repeatable procedure Diagnostic and therapeutical procedure Reduced costs Reduced morbidity Distinguish benign effusion from malignant Reduce time of diagnostic and molecular profiling Photography of cancer heterogeneity Monitoring of treatment response Monitoring changing in genomic landscape Its composition could inform about patient prognosis

## Data Availability

No new data were created or analyzed in this study. Data sharing is not applicable to this article.
